# Geographic variation in the costs of medical care for people living with HIV in British Columbia, Canada

**DOI:** 10.1186/s12913-019-4391-8

**Published:** 2019-09-03

**Authors:** Benjamin Enns, Jeong Eun Min, Dimitra Panagiotoglou, Julio S. G. Montaner, Bohdan Nosyk, On behalf of the STOP HIV/AIDS study group, Rolando Barrios, Patty Daly, Reka Gustafson, Perry R. W. Kendall, Gina McGowan, Irene Day, Kate Heath, Robert S. Hogg, Julio S. G. Montaner, Bohdan Nosyk

**Affiliations:** 1 BC Centre for Excellence in HIV/AIDS, St. Paul’s Hospital, 613-1081 Burrard St, Vancouver, BC V6Z 1Y6 Canada; 2 Faculty of Medicine, Department of Epidemiology, Biostatistics and Occupational Health, McGill University, 1020 Pine Avenue West, Montreal, QC H3A 1A2 Canada; 3 Division of AIDS, Department of Medicine, University of British Columbia, 667-1081 Burrard Street, Vancouver, BC V6Z 1Y6 Canada; 4 Faculty of Health Sciences, Simon Fraser University, Blusson Hall, Room 11300, 8888 University Drive, Burnaby, BC V5A 1S6 Canada

**Keywords:** Medical costs, HIV, HIV costs, Regional costs, Medical utilization

## Abstract

**Background:**

Regional variation in medical care costs can indicate heterogeneity in clinical practice, inequities in access, or inefficiencies in service delivery. We aimed to estimate regional variation in medical costs for people living with HIV (PLHIV), adjusting for demographics and case-mix.

**Methods:**

We conducted a retrospective cohort study using linked health administrative databases of PLHIV, from 2010 to 2014, in British Columbia (BC), Canada. Quarterly health care costs (2018 CAD) were derived from inpatient, outpatient, prescription drugs, antiretroviral therapy (ART), and HIV diagnostics. We used a two-part model with a logit link for the probability of incurring costs, and a log link and gamma distribution for observations with positive costs. We also estimated quarterly utilization rates for hospitalization-, physician billing- and prescription drug-days. Primary variables were indicators of individuals’ Health Service Delivery Area (HSDA). We adjusted cost and utilization estimates for demographic characteristics, HIV-disease progression, and comorbidities.

**Results:**

Our cohort included 9577 PLHIV (median age 45.5 years, 80% male). Adjusted total quarterly costs for all 16 HSDAs were within 20% of the provincial mean, 8/16 for hospitalization costs, 16/16 for physician billing costs and 10/16 for prescription drug costs. Northern Interior and Northeast HSDAs had 38 and 44% lower quarterly non-ART prescription drug costs, and 2 and 5% higher quarterly inpatient costs, respectively.

**Conclusions:**

We observed limited variation in medical care costs and utilization among PLHIV in BC. However, lower levels of outpatient care and higher levels of inpatient care indicate possible barriers to accessing care among PLHIV in the most rural regions of the province.

**Electronic supplementary material:**

The online version of this article (10.1186/s12913-019-4391-8) contains supplementary material, which is available to authorized users.

## Background

Regional variation in health care costs after adjustment for demographic and clinical factors can be indicative of inequities or a lack of well-defined clinical practice [1, 2]. This topic has been the subject of considerable research in the United States and elsewhere [1, 3–5], primarily to facilitate performance-based reimbursement or identify potentially inefficient health care providers [1]. While many of the drivers of regional disparities (which include variation in insurance coverage, multiple care providers, and other potential barriers to access [6, 7]) may not be applicable in single-payer systems [8], regional variation was nonetheless identified among individuals with gastric cancer in Ontario [2], as well as among stroke patients in Alberta, though this was found to be diminishing over time [9]. In British Columbia, variation in healthcare costs was found to be modest in the general population after adjusting for individuals’ characteristics [8].

Like other settings with concentrated HIV epidemics, there is substantial regional heterogeneity in the HIV epidemic within British Columbia (BC), demonstrated by differences in rates of new diagnoses, mortality rates, and availability of HIV-testing and harm reduction services [10, 11]. Health Authorities (HA) and Health Service Delivery Areas (HSDA) within the province are regional administrative boundaries by which health care funding is allocated and decisions are made (Additional file 1: Figure S1). HSDAs are heterogeneous in population density, with areas ranging from less than 0.5, to more than 800 persons per square kilometer [12]. Furthermore, 88% of all combination antiretroviral therapy (ART)-prescribing physicians in BC practice in urban areas with specialists and more experienced ART-prescribing physicians easier to access [13]. Regional disparities in HIV care among BC’s health regions have been consistently documented [10, 14, 15]. Most notably, ART engagement and viral suppression rates in 2016 were 7 and 23 percentage points lower, respectively, in the Northern HA compared to Vancouver Coastal HA which is home to over 50% of people living with HIV (PLHIV) in BC and 25% of the provincial population [16, 17].

Targeting geographic regions for structural improvements is more straightforward for health policy makers, given the organization of health care delivery, and potential barriers to access driven by geographic factors [12, 18, 19]. The BC Ministry of Health established HA-specific targets for the reach of HIV testing and access to ART, among other endpoints [11]. Disbursement of funds to meet these goals, as well as other supports, such as distribution of harm reduction supplies, is allotted by HA [11]. The province also has a number of programs targeting regional inequities in care, including rural retention programs which provide incentives for physicians to work in rural and remote communities, as well as expansion of telehealth services, which provide consultations to individuals in remote areas [12, 18]. Despite these concerted efforts to address regional variation in clinical practice and access to HIV care, it is unclear whether disparities in healthcare delivery persist. Analyzing the costs of the key forms of medical care for PLHIV across the province may help guide targeted intervention and thus support a localized response to the HIV/AIDS epidemic in BC.

Our aim was to quantify regional variation in medical care costs for PLHIV. We generated disaggregated estimates of costs and utilization rates for hospitalizations, physician billings and drug dispensations (both with and without the costs of ART medications) to identify potential associations between components, particularly in regions where lower levels of outpatient care could be leading to higher levels of inpatient utilization.

## Methods

### Study design and data sources

We conducted a retrospective cohort study using linked, provincial health administrative databases and disease registries, including the BC Centre for Excellence in HIV/AIDS drug treatment program and virology registries (antiretroviral dispensations, plasma viral load (pVL) and CD4 tests) [20], the BC Centre for Disease Control HIV testing database (HIV diagnosis/risk group classification) [21], the Medical Services Plan (MSP) database (physician billing records) [22], the Discharge Abstract database (hospitalizations) [23], the BC PharmaNet database (non-antiretroviral drug dispensations) [24], and the BC Vital statistics database (deaths) [25]. Our data set comprised all HIV positive individuals identified within these databases, observed for at least two quarters between January 1, 2010 and March 31, 2014. Details regarding databases and cohort construction have been published elsewhere [17]. Our data included quarterly observations from HIV diagnosis to death, administrative loss to follow-up (no record of health service use for at least 18 months), or censorship as of March 31, 2014. We chose quarterly observations according to CD4 and pVL monitoring guidelines from the International Antiviral Society–USA (IAS-USA) [26]. The health care setting featured government-funded, single-payer care, covering inpatient and outpatient care, selected prescription drugs, laboratory monitoring, and antiretroviral medications.

### Study measures and variable creation

Our primary outcome variable was the quarterly cost of medical care; calculated as the sum of inpatient, outpatient (physician fee-for-service billing claims), prescription drug costs (ART and non-ART), and diagnostic testing costs for each individual in our cohort. We estimated medical costs for PLHIV including the costs of ART medication, excluding ART medication, as well as costs of ART medication only for those receiving ART. We presented each of these analyses separately, as differential rates of ART uptake across HSDAs, combined with the high relative costs of antiretroviral medication, could potentially obscure differences in health care costs that indicated divergence from clinical practice standards or higher intensity of service provision among PLHIV. The methods for deriving these costs have been described previously [27, 28]. We adjusted all costs to 2018 Canadian Dollars (CAD), using the Canadian Consumer Price Index.

To support our primary analysis, and to provide further context as to which health care components showed greater regional differences among PLHIV, we estimated costs and utilization rates for hospitalizations, physician billing records and non-ART prescription drugs, respectively. We defined utilization outcomes as hospitalization days (days with an inpatient record), physician billing days (number of days in which an individual had at least one physician billing contact) - chosen to reflect the frequency of contacts rather than the number of individual MSP billing line items - and drug days prescribed (the total of all prescription days in a given quarter).

### Geographical health regions

Our primary independent variables were a set of quarterly indicator variables for an individual’s primary HSDA. We assigned one representative HSDA to each person in each quarter, depending on where an individual accessed health care services. We assigned time-varying indicators of HSDA due to high rates of intra-provincial migration for treatment among PLHIV [29, 30]. When an individual accessed health services in more than one HSDA in a quarter, we selected the HSDA in which an individual recorded the highest frequency of health care contacts as the representative HSDA.

We adjusted for a range of clinical factors, including measures of HIV disease progression (CD4 < 200 cells/μL; 200–499 cells/μL; ≥500 cells/μL; unmeasured) and ART status (ART-naïve, on-ART, and off-ART post-initiation (ART-dropout)). Additionally, we included the area under the log_10_ plasma viral load curve (AUC pVL) for 12-months prior to the start of the quarter (< 2.7; 2.7–2.99; 3–3.49; ≥3.5; unmeasured) to capture individuals’ cumulative viral load over the previous 12-months. CD4 counts were taken from the most recent test prior to the start of the quarter, with previous test results carried forward for any missing observations, otherwise CD4 count was classified as unmeasured. AUC pVL measurements were also carried forward until the next non-missing observation, or the end of follow-up, otherwise AUC pVL was classified as unmeasured.

Demographic control variables included: age, sex, calendar year, transmission risk group, including: men who have sex with men (MSM); people who inject drugs (PWID); MSM who inject drugs (MWID); heterosexual/other (HET), year of diagnosis, grouped as: pre-1996; 1997–99; 2000–03; 2004–07; 2008–14, and two separate measures of medical comorbidity, the drug prescription-based Chronic Disease Score (CDS) [31], and the Charlson Comorbidity Index (CCI) [32], based on hospital records, calculated as a moving average for the year prior to quarter start date. Finally, to account for individuals potentially re-locating for specialized, high-cost health care services, we included a variable indicating if an individual had moved in the previous 12 months, based on changes to annually updated MSP-registered billing addresses.

### Analysis

To estimate quarterly health care costs, we fit a two-part generalized linear model (GLM) with logit link and binomial distribution for the probability of an individual incurring any costs, and a log link with gamma distribution for observations with non-zero costs [33]. We estimated costs per person-quarter by HSDA, holding all other covariates fixed at their overall sample means.

We estimated utilization rates for each component, using a two-part generalized linear model (GLM) with logit link and binomial distribution for the probability of an individual having any utilization, and a log link and negative binomial distribution for observations with non-zero utilization.

### Sensitivity analysis

We repeated our primary analysis using representative HSDAs assigned by highest cost, rather than highest frequency of visits, in a given quarter. We conducted additional sensitivity analysis removing costs for services received through the Provincial Health Services Authority (which provides specialized health services to the entire province), which were otherwise assigned to an individual’s representative HSDA, to determine if estimates for some HSDAs were disproportionately affected.

We created our analytical sample in SAS 9.4, and conducted statistical analysis in Stata 14.1.

## Results

Our sample included 9577 individuals, with 140,137 person-quarters of observation between January 1, 2010 and March 31, 2014. Our cohort was 20% female, with a median age of 45.5 years at baseline, 22.9% were known to be PWID and 28.5% MSM (Table 1). Of the 16 total HSDAs, the largest served 7068 different individuals (73.8% of all individuals in our sample), the smallest served 75 individuals, and 66.7% of individuals received services in more than one primary HSDA over the study period. Across HSDAs, the proportion of female person-quarters ranged from 14.2–42.0%, MSM person-quarters ranged from 3.8–44.9% and PWID person-quarters ranged from 18.1–59.0%. Individuals were on ART in 74.6% of all person-quarters in our sample, Vancouver had the highest percentage (81.8%), and the Northeast had the lowest (49.4%) (Table 2).

**Table 1:** Summary statistics on individuals at baseline and summary statistics for medical costs by component

		Individuals (n)		(%)	
Baseline sample characteristics (*n* = 9577)					
Female		1917		20%	
Period of diagnosis:					
*< 1996*		1297		13.5%	
*1997–1999*		2186		22.8%	
*2000–2003*		1657		17.3%	
*2004–2007*		1789		18.7%	
*2008–2014*		2648		27.6%	
HIV risk group:					
*PWID*		2363		24.7%	
*MSM*		3116		32.5%	
*MWID*		753		7.9%	
*HET*^a^		3345		34.9%	
Age (median)		45.5		–	
	Cost Component				
	Hospitalization	Physician Billings	Non-ART Prescriptions	ART Prescriptions	Total Costs
Zero-cost observations (%)	94.6%	13.4%	34.8%	27.6%	5.0%
Positive-cost observations					
*Mean*	$14,273	$530	$711	$4741	$5712
*SD*	($19,635)	($946)	($1376)	($1260)	($7005)
*Skewness*	4.2	7.7	9.4	1.9	10.7
*Kurtosis*	28.8	99.1	202.6	7	213.5
*50th percentile*	$7629	$287	$239	$4689	$5262
*99th percentile*	$96,497	$4638	$5519	$9884	$31,362

*ART* Antiretroviral Therapy, *PWID* People who inject drugs, *MSM* Men who have sex with men, *HET* Heterosexual

^a^−Includes those in other and unknown HIV risk groups

**Table 2:** Descriptive statistics of selected time-varying covariates by HSDA

	Individuals^a^	Observations	Percentage of person-quarters (observations)						
				Risk group^b^			ART status		
			Female	MSM	PWID	MWID	on ART	off ART	ART-naïve
Overall	9577	140,137	20.1%	33.2%	25.6%	8.4%	74.6%	19.4%	5.9%
Interior Health									
*East Kootenay*	75	443	33.2%	23.0%	18.5%	0.7%	52.8%	43.1%	4.1%
*Kootenay Boundary*	93	794	35.4%	21.9%	37.0%	2.6%	75.3%	17.1%	7.6%
*Okanagan*	546	4913	23.6%	22.0%	32.0%	8.7%	72.8%	22.1%	5.1%
*Thompson Cariboo*	305	2668	25.6%	17.5%	34.1%	3.8%	53.6%	40.6%	5.8%
Fraser Health									
*Fraser East*	573	3191	35.6%	16.1%	32.8%	6.5%	59.9%	36.2%	3.9%
*Fraser North*	3126	14,756	26.8%	14.6%	35.7%	6.4%	61.9%	32.3%	5.8%
*Fraser South*	1569	8309	34.5%	15.5%	39.1%	4.5%	65.4%	27.6%	7.1%
Vancouver Coastal Health									
*Richmond*	270	1355	28.6%	19.2%	25.8%	4.0%	62.1%	29.0%	8.9%
*Vancouver*	7068	80,964	14.2%	44.9%	18.1%	10.0%	81.8%	12.7%	5.5%
*North Shore/Coast Garibaldi*	497	2586	22.0%	23.6%	26.5%	4.8%	62.0%	31.4%	6.6%
Island Health									
*South Vancouver Island*	1509	10,869	19.1%	22.1%	33.7%	8.1%	72.1%	20.4%	7.5%
*Central Vancouver Island*	423	3304	30.3%	19.5%	33.5%	7.9%	68.8%	23.9%	7.4%
*North Vancouver Island*	168	1499	42.0%	8.7%	43.2%	8.7%	65.6%	28.2%	6.2%
Northern Health									
*Northwest*	146	1179	40.2%	3.8%	59.0%	3.7%	53.8%	36.8%	9.4%
*Northern Interior*	335	2734	38.7%	10.8%	51.8%	2.3%	59.2%	33.6%	7.0%
*Northeast*	85	573	35.1%	7.5%	23.7%	5.4%	49.4%	44.0%	6.6%

*ART* Antiretroviral Therapy, *MSM* Men who have sex with men, *PWID* Person who injects drugs, *MWID* MSM who inject drugs

^a^Number of individuals by HSDA is the number of unique individuals ever in a particular HSDA over the course of the study. Given that individuals can appear in multiple HSDAs over time, the sum of individuals by HSDA is higher than the total number of individuals

^b^Remaining percentage includes heterosexual, other and unknown risk

**Fig. 1:**
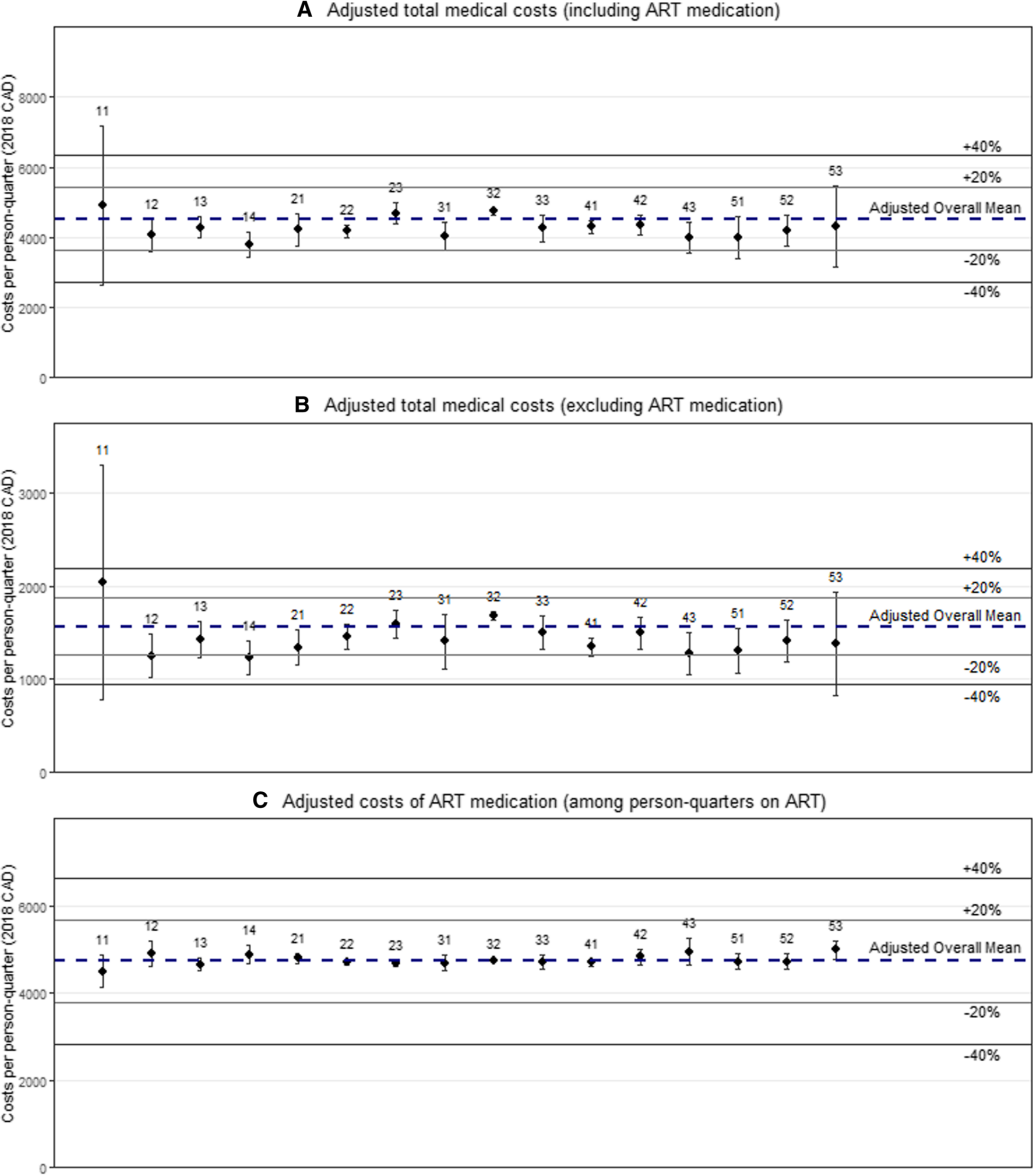
Multiple regression results for adjusted quarterly medical care costs, including and excluding ART medication, per person-quarter by Health Service Delivery Area. Panel **a** shows results for total medical costs, Panel **b** shows results for costs excluding ART medication, and Panel **c** shows results for ART medication costs only among those receiving ART. Legend: 11 – East Kootenay; 12 – Kootenay Boundary; 13 – Okanagan; 14 – Thompson Cariboo Shuswap; 21 – Fraser East; 22 – Fraser North; 23 – Fraser South; 31 – Richmond; 32 – Vancouver; 33 – North Shore/Coast Garibaldi; 41 – South Vancouver Island; 42 – Central Vancouver Island; 43 – North Vancouver Island; 51 – Northwest; 52 – Northern Interior; 53 - Northeast. Covariate adjustment included: age, gender, era of diagnosis, calendar year, moved in past 12 months, ART-status, CD4 cell count, pVL, chronic disease score, Charlson comorbidity index, HIV risk group

Quarterly medical costs, adjusting for clinical and demographic factors and including ART, ranged from $3775 [$3407, $4143] in Thompson Cariboo, to $4912 [$2636, $7188] in East Kootenay, with all 16 HSDAs falling within 20% of the overall provincial mean (Fig.1a & Additional file 1: Table S1). Adjusted quarterly costs, excluding ART, ranged from $1232 [$1052, $1412] in Thompson Cariboo to $2043 [$785, $3301] in East Kootenay, with 13/16 HSDAs falling within 20% of the overall provincial mean, and all 16 within 40% (Fig.1b & Additional file 1: Table S1). Adjusted costs of ART medication per person-quarter on ART, ranged from $4501 [$4131, $4871] for PLHIV in East Kootenay to $4982 [$4766, $5198] for PLHIV in the Northeast HSDA, and all HSDAs were within 6% of the provincial mean (Fig.1c & Additional file 1: Table S1).

**Fig. 2:**
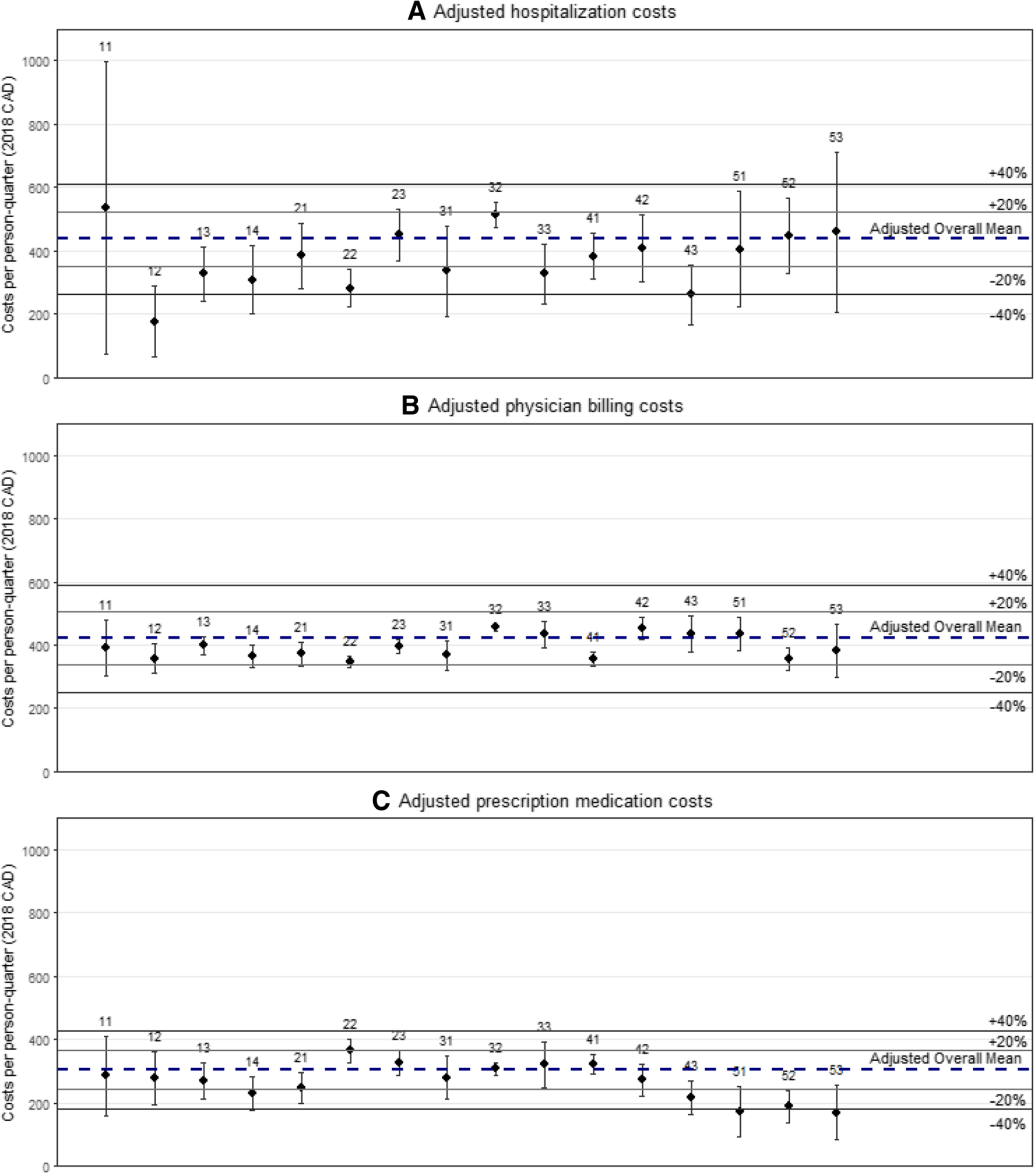
Multiple regression results for adjusted quarterly inpatient, physician billing and prescription drug costs per person-quarter by Health Service Delivery Area. Panel **a** shows results for only hospitalization costs, Panel **b** shows results for only physician billing costs and Panel **c** shows results for only prescription medication costs. Legend: 11 – East Kootenay; 12 – Kootenay Boundary; 13 – Okanagan; 14 – Thompson Cariboo Shuswap; 21 – Fraser East; 22 – Fraser North; 23 – Fraser South; 31 – Richmond; 32 – Vancouver; 33 – North Shore/Coast Garibaldi; 41 – South Vancouver Island; 42 – Central Vancouver Island; 43 – North Vancouver Island; 51 – Northwest; 52 – Northern Interior; 53 – Northeast. Covariate adjustment included: age, gender, era of diagnosis, calendar year, moved in past 12 months, ART-status, CD4 cell count, pVL, chronic disease score, Charlson comorbidity index, HIV risk group

**Fig. 3:**
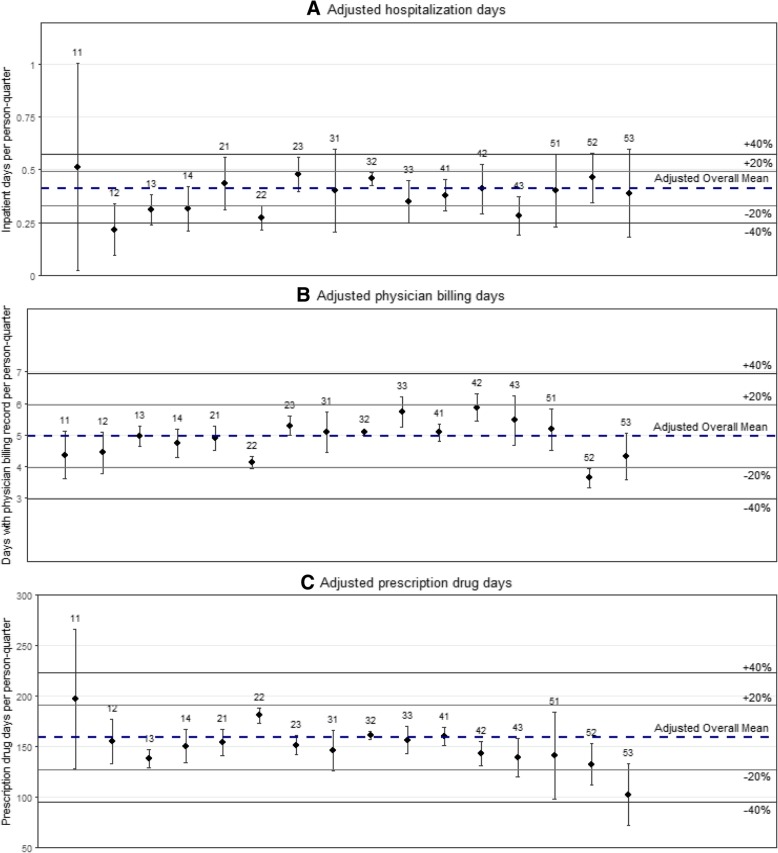
Multiple regression results for adjusted quarterly inpatient, physician billing and prescription drug utilization rates per person-quarter by Health Service Delivery Area. Panel **a** shows results for only hospitalization days, Panel **b** shows results for only physician billing days and Panel **c** shows results for only prescription drug days. Legend: 11 – East Kootenay; 12 – Kootenay Boundary; 13 – Okanagan; 14 – Thompson Cariboo Shuswap; 21 – Fraser East; 22 – Fraser North; 23 – Fraser South; 31 – Richmond; 32 – Vancouver; 33 – North Shore/Coast Garibaldi; 41 – South Vancouver Island; 42 – Central Vancouver Island; 43 – North Vancouver Island; 51 – Northwest; 52 – Northern Interior; 53 – Northeast. Covariate adjustment included: age, gender, era of diagnosis, calendar year, moved in past 12 months, ART-status, CD4 cell count, pVL, chronic disease score, Charlson comorbidity index, HIV risk group

Adjusted mean hospitalization days ranged from 0.22 [0.09, 0.34] in Kootenay Boundary to 0.51 [0.02, 1.01] per person-quarter in East Kootenay, and 10/16 HSDAs were within 20% of the overall mean (Fig. 3a & Additional file 1: Table S3). Adjusted mean hospitalization costs ranged from $176 [$62, $289] in North Vancouver Island, to $535 [$73, $996] in East Kootenay. Half of all HSDAs were within 20% of the overall mean, while hospitalization costs in Kootenay Boundary, North Vancouver Island, and Fraser were more than 30% lower than the provincial mean (Fig. 2a & Additional file 1: Table S2).

Adjusted mean days with a physician billing record ranged from 3.65 [3.34, 3.95] for PLHIV in the Northern Interior, to 5.87 [5.43, 6.30] per person-quarter for PLHIV in Central Vancouver Island, and 15/16 HSDAs were within 20% of the adjusted overall mean (Fig. 3b & Additional file 1: Table S3). Adjusted mean quarterly physician billing costs ranged from $348 [$332, $363] in Fraser North, to $455 [$446, $465] in Vancouver, and all 16 HSDAs were within 20% of the adjusted overall mean (Fig. 2b & Additional file 1: Table S2).

Adjusted prescription drug days ranged from 103 [73, 133] in the Northeast, to 197 [128, 266] per person-quarter in East Kootenay, and 14/16 HSDAs were within 20% of the adjusted overall mean (Fig. 3c & Additional file 1: Table S3). Adjusted quarterly prescription drug costs ranged from $170 [$83, $257] in Northeast, to $366 [$328, $403] in Fraser North, and 10/16 of HSDAs were within 20% of the adjusted overall mean (Fig. 2c & Additional file 1: Table S2). Furthermore, adjusted prescription drug costs were 43, 38 and 44% lower than the overall average in the Northwest, Northern Interior and Northeast HSDAs, respectively (Fig. 2c & Additional file 1: Table S2).

Assigning HSDA by cost rather than frequency of contacts produced minor changes in estimates, with the largest change occurring in the Northwest HSDA, where adjusted quarterly costs were reduced to $3798 [$3181, $4415] from $3989 [$3377, $4601]. Removing PHSA costs from our analysis reduced adjusted quarterly costs to $3909 [$3182, $4636], from $4311 [$3151, $5471] in the Northeast HSDA, and all other changes were smaller in magnitude (Additional file 1: Table S4).

## Discussion

Our results showed moderate regional variation in medical costs for PLHIV in British Columbia, adjusting for demographic variables and case-mix. For estimates of total costs, including the costs of ART medication, costs per person-quarter in all 16 HSDAs were within 20% of the overall provincial mean, and cost differences between the highest and lowest cost HSDAs were $948 [$571, $1325] (Fig.1a), equivalent to 21% of the overall provincial mean. For estimates of total costs, excluding costs of ART medications, 13 of 16 HSDAs in British Columbia were within 20% of the overall mean, and costs for PLHIV in the highest cost HSDA were $444 [$257, $630] higher than the lowest cost HSDA per person-quarter, a difference equivalent to 28% of the overall provincial mean. While ART uptake rates differed, ART costs among PLHIV who were receiving ART were homogeneous across HSDAs.

Variation in annual health care costs in the general population was previously estimated to be 21% between the highest- and lowest-spending HSDAs in British Columbia, after adjustment for age, sex, aggregated diagnosis group, and health service environment [8]. In the United States, over 31% of geographic variation in prostate cancer care costs among Medicare recipients was not explained by covariate adjustment, including age, race, year of diagnosis, rural residence, income, other comorbidities, in addition to detailed adjustment for treatment intensity, ancillary procedures, and treatment modalities [5]. Among PLHIV in the United States, mean Medicaid payments were more than double for individuals in New York, compared to Massachusetts [34]. Given that our study cohort comprised a high-cost, disease-specific subset of the general population, our results suggested that the regional variation observed in medical costs among PLHIV in British Columbia, with and without ART medication costs, was comparable to the general population. As our data did not include individual-level socioeconomic variables such as income, or regional measures of health service environments, such as the presence or absence of large hospitals, adjustment for these factors could have further attenuated regional cost differences.

That we observed moderate regional differences in non-ART medical costs among PLHIV, after adjusting for demographics, disease progression and other comorbidities, suggests that standards of care were not substantially different across regions for PLHIV. This may be due, in part, to the maturity of the HIV care infrastructure in the province. Previous studies of inter-provincial variation in HIV testing rates have suggested that coordinating all HIV care in the province through a single center may produce better outcomes, by allowing for closer monitoring of PLHIV and better control over standards of care [35], and regional variation tends to be larger when well-defined standards of care and guidelines are absent [2]. Furthermore, the small variation in ART costs among those receiving ART, is likely a result of the evolution of ART treatment, with simpler and safer regimens [36], easing some of the burden on rural health regions by lessening the requirement for highly-specialized clinics and physicians to treat PLHIV effectively [30]. The expansion of the STOP HIV/AIDS program to the entire province in 2012 instituted comprehensive monitoring of PLHIV from testing through care linkage, as well as harmonizing all aspects of HIV care [14, 37], including elimination of pre-test counseling, simplified treatment and initiation protocols, and financial incentives for HIV-related care visits [38]. All of these factors, designed to simplify delivery of HIV testing and care, may have contributed to the relatively low levels of adjusted regional variation in health care costs among PLHIV in British Columbia.

In our analysis of the individual components of medical costs and utilization, it was noteworthy that PLHIV in Northern Interior and Northeast HSDAs had 38 and 44% lower prescription drug costs, 15.3 and 9.1% lower physician billing costs, and 2.3 and 4.9% higher hospitalization costs, respectively. PLHIV in both HSDAs were below the overall mean in physician billing days, as well as prescription drug days. Furthermore, PLHIV in these HSDAs had lower levels of ART uptake than the provincial average (Table 2), and if individuals faced barriers in accessing prescription medication or primary care due to geographic remoteness, or other factors, this may have been reflected in higher inpatient utilization [39, 40].

There is a substantial literature examining the interaction between the use of inpatient, outpatient, and prescription drug utilization. Previous studies have examined changes to insurance coverage or increased deductibles resulting in lower prescription drug use [41–44], the effect of poor adherence to medication on other health care utilization [45, 46], as well as the effect of increased prescription fills on inpatient costs [47]. In British Columbia, Li et al. (2007) found that increased cost sharing reduced prescription drug utilization and led to increased physician visits among seniors [44]. Furthermore, Law et al. (2017) found that the addition of a small deductible to health plans already requiring co-payment was not associated with reductions in overall prescription drug use or increases in non-pharmaceutical health care utilization [43]. Among PLHIV in the United States, those with higher outpatient utilization had higher inpatient admissions, however, those with no outpatient visits over a 3-month period had significantly higher inpatient admission rates, suggesting that PLHIV with no visits were at higher risk for hospitalization due to a lack of clinical monitoring [48]. While our study was not designed to estimate the causal relationship between changes in outpatient health care utilization and acute care utilization or costs, this could nonetheless be indicative of an association between lower levels of outpatient care and higher levels of inpatient care.

### Limitations

This analysis had several limitations. First, HSDA assignment was subject to some degree of misclassification due to the possibility of individuals receiving care in multiple HSDAs in a given quarter or missing information on HSDA, however, our results were robust to alternative HSDA assignment methods in sensitivity analysis (Additional file 1: Table S4). Second, PHSA costs were assigned to an individual’s representative HSDA, and could potentially have affected some regions disproportionately, however, our HSDA-specific cost estimates were robust to the removal of these costs (Additional file 1: Table S4). Furthermore, due to a small number of observations, results from the East Kootenay HSDA should be interpreted with caution. Finally, as with all non-experimental studies, we cannot rule out the possibility that our coefficient estimates may have been subject to some degree of bias from unmeasured confounding factors [49]. It is likely that some degree of the variation in regional costs could be attributable to incomplete case-mix adjustment. While our study included detailed information on HIV treatment and disease progression, it is possible that the prevalence and severity of comorbid conditions were not fully accounted for. Finally, our goal was to estimate variation among HSDAs in costs and utilization rates, and we did not examine the association between health care costs and quality of care, clinical outcomes or long-term spending by region. Further investigation is needed into the association between utilization rates of different health care components, particularly in rural and remote regions, to determine if lack of access, socioeconomic factors or other barriers to care are leading to higher hospitalization utilization and costs, or other adverse outcomes.

## Conclusions

We estimated the magnitude of geographic variation in medical costs and utilization rates among PLHIV in British Columbia from 2010 to 2014, using linked, individual-level data to characterize regional differences. Despite substantial regional differences in demographic composition, ART uptake, and HIV care engagement, we found that variation in medical costs among PLHIV, both including and excluding ART medication, was comparable to the overall population of British Columbia, adjusting for demographic variables and case-mix. While there was generally modest variation among HSDAs for hospitalizations, physician billings and non-ART prescriptions, our results indicated possible substitution between outpatient care and inpatient care among PLHIV in the most rural regions of the province.

## Additional files

Additional file 1: This supplement contains the data plotted in Figs. 1, 2 and 3 of the manuscript, as well as results from sensitivity analysis. It also includes a map of Health Service Delivery Areas in British Columbia. (DOCX 532 kb)

## Data Availability

The data that support the findings of this study are available from Seek And Treat For Optimal Prevention Of HIV/AIDS® (STOP HIV/AIDS®) but restrictions apply to the availability of these data, which were used under license for the current study, and so are not publicly available. Data are however available from the authors upon reasonable request and with permission of Seek And Treat For Optimal Prevention Of HIV/AIDS® (STOP HIV/AIDS®).

## Abbreviations

AIDS: Acquired immunodeficiency syndrome
ART: Antiretroviral therapy
AUC: Area under the curve
BC: British Columbia
CAD: Canadian dollars
CCI: Charlson comorbidity index
CDS: Chronic disease score
GLM: Generalized linear model
HA: Health authority
HET: Heterosexual
HIV: Human immunodeficiency virus
HSDA: Health service delivery area
IAS-USA: International Antiviral Society–USA
MSM: Men who have sex with men
MSP: Medical services plan
MWID: Men who have sex with men and inject drugs
PLHIV: People living with HIV
pVL: Plasma viral load
PWID: People who inject drugs
