# Supplementary material for: Geographic variation in the costs of medical care for people living with HIV in British Columbia, Canada
Source: BMC Health Serv Res. 2019 Sep 3;19:626. doi: 10.1186/s12913-019-4391-8 (PMC6724338; doi:10.1186/s12913-019-4391-8)
Supplement: Supplementary file 1 — This supplement contains the data plotted in Figs. 1, 2 and 3 of the manuscript, as well as results from sensitivity analysis. It also includes a map of Health Service Delivery Areas in British Columbia. (DOCX 532 kb) [file 12913_2019_4391_MOESM1_ESM.docx]

**Additional file**

**Geographic variation in the costs of medical care for people living with HIV in British Columbia, Canada**

Benjamin Enns, MA [1], Jeong Eun Min, MSc [1], Dimitra Panagiotoglou, PhD [2], Julio S.G. Montaner, MD [1,3], Bohdan Nosyk, PhD [1,4], **on behalf of the STOP HIV/AIDS study group.**

1. Health Economic Research Unit at the British Columbia Centre for Excellence in HIV/AIDS; 2. Faculty of Medicine, Department of Epidemiology, Biostatistics and Occupational Health, McGill University; 3. Division of AIDS, Department of Medicine, University of British Columbia, 4. Faculty of Health Sciences, Simon Fraser University.

**Table S1. Multiple regression results for adjusted total quarterly medical costs per person-quarter by Health Service Delivery Area**

|  |  |  |  |  |  |  |
| --- | --- | --- | --- | --- | --- | --- |
|  | **(1)** | | **(2)** | | **(3)** | |
| Interior Health |  | [95% C.I.] |  | [95% C.I.] |  | [95% C.I.] |
| *East Kootenay* | $4912 | [$2636, $7188] | $2043 | [$785, $3301] | $4501 | [$4131, $4871] |
| *Kootenay Boundary* | $4048 | [$3580, $4516] | $1247 | [$1014, $1480] | $4894 | [$4610, $5178] |
| *Okanagan* | $4274 | [$3976, $4572] | $1423 | [$1225, $1621] | $4656 | [$4517, $4795] |
| *Thompson Cariboo* | $3775 | [$3407, $4143] | $1232 | [$1052, $1412] | $4885 | [$4673, $5097] |
| Fraser Health |  |  |  |  |  |  |
| *Fraser East* | $4204 | [$3749, $4659] | $1339 | [$1153, $1525] | $4795 | [$4693, $4897] |
| *Fraser North* | $4170 | [$3980, $4360] | $1452 | [$1319, $1585] | $4706 | [$4653, $4759] |
| *Fraser South* | $4677 | [$4375, $4979] | $1584 | [$1435, $1733] | $4686 | [$4619, $4753] |
| Vancouver Coastal Health |  |  |  |  |  |  |
| *Richmond* | $4028 | [$3624, $4432] | $1404 | [$1112, $1696] | $4694 | [$4531, $4857] |
| *Vancouver* | $4722 | [$4630, $4814] | $1676 | [$1629, $1723] | $4728 | [$4695, $4761] |
| *North Shore/Coast Garibaldi* | $4245 | [$3871, $4619] | $1497 | [$1317, $1677] | $4706 | [$4551, $4861] |
| Island Health |  |  |  |  |  |  |
| *South Vancouver Island* | $4294 | [$4110, $4478] | $1345 | [$1251, $1439] | $4695 | [$4615, $4775] |
| *Central Vancouver Island* | $4346 | [$4068, $4624] | $1495 | [$1323, $1667] | $4833 | [$4657, $5009] |
| *North Vancouver Island* | $3975 | [$3538, $4412] | $1275 | [$1054, $1496] | $4942 | [$4638, $5246] |
| Northern Health |  |  |  |  |  |  |
| *Northwest* | $3989 | [$3377, $4601] | $1302 | [$1065, $1539] | $4720 | [$4546, $4894] |
| *Northern Interior* | $4184 | [$3751, $4617] | $1406 | [$1179, $1633] | $4724 | [$4561, $4887] |
| *Northeast* | $4311 | [$3151, $5471] | $1378 | [$817, $1939] | $4982 | [$4766, $5198] |
| *(1) - Baseline model including all costs; **(2) - Baseline model excluding ART costs; ***(3) - Model including only ART costs among PLHIV on ART Covariate adjustment for all models included: age, gender, era of diagnosis, calendar year, moved in past 12 months, ART-status, CD4 cell count, pVL, chronic disease score, Charlson comorbidity index, HIV risk group. | | | | | | |
|  |  |  |  |  |  |  |
|  |  |  |  |  |  |  |
|  |  |  |  |  |  |  |

**Table S2. Multiple regression results for adjusted quarterly inpatient, physician billing and non-ART prescription drug costs, per person-quarter by Health Service Delivery Area**

|  |  |  |  |  |  |  |
| --- | --- | --- | --- | --- | --- | --- |
|  | **(1)** | | **(2)** | | **(3)** | |
| Interior Health |  | [95% C.I.] |  | [95% C.I.] |  | [95% C.I.] |
| *East Kootenay* | $535 | [$73, $996] | $391 | [$305, $477] | $285 | [$161, $409] |
| *Kootenay Boundary* | $176 | [$62, $289] | $358 | [$312, $403] | $277 | [$193, $361] |
| *Okanagan* | $327 | [$242, $411] | $399 | [$371, $427] | $270 | [$212, $328] |
| *Thompson Cariboo* | $308 | [$199, $416] | $365 | [$329, $401] | $229 | [$177, $281] |
| Fraser Health |  |  |  |  |  |  |
| *Fraser East* | $384 | [$279, $489] | $374 | [$336, $411] | $248 | [$198, $298] |
| *Fraser North* | $281 | [$221, $342] | $348 | [$332, $363] | $366 | [$328, $403] |
| *Fraser South* | $449 | [$369, $530] | $395 | [$372, $419] | $325 | [$286, $364] |
| Vancouver Coastal Health |  |  |  |  |  |  |
| *Richmond* | $336 | [$194, $478] | $367 | [$323, $411] | $280 | [$213, $348] |
| *Vancouver* | $513 | [$474, $552] | $455 | [$446, $465] | $308 | [$287, $329] |
| *North Shore/Coast Garibaldi* | $326 | [$233, $420] | $434 | [$391, $477] | $320 | [$249, $392] |
| Island Health |  |  |  |  |  |  |
| *South Vancouver Island* | $383 | [$310, $457] | $357 | [$336, $378] | $321 | [$290, $353] |
| *Central Vancouver Island* | $407 | [$300, $514] | $454 | [$420, $489] | $272 | [$221, $324] |
| *North Vancouver Island* | $260 | [$167, $353] | $435 | [$376, $494] | $216 | [$164, $268] |
| Northern Health |  |  |  |  |  |  |
| *Northwest* | $404 | [$223, $586] | $434 | [$381, $488] | $173 | [$95, $252] |
| *Northern Interior* | $447 | [$328, $565] | $356 | [$321, $391] | $188 | [$137, $239] |
| *Northeast* | $458 | [$205, $712] | $382 | [$298, $466] | $170 | [$83, $257] |
| *(1) - Adjusted hospitalization costs per person-quarter; **(2) - Adjusted physician billing costs per person-quarter; ***(3) - Adjusted non-ART prescription drug costs per person-quarter Covariate adjustment for all models included: age, gender, era of diagnosis, calendar year, moved in past 12 months, ART-status, CD4 cell count, pVL, chronic disease score, Charlson comorbidity index, HIV risk group. | | | | | | |
|  |  |  |  |  |  |  |
|  |  |  |  |  |  |  |
|  |  |  |  |  |  |  |

**Table S3. Multiple regression results for adjusted quarterly inpatient, physician billing and prescription drug utilization rates per person-quarter by Health Service Delivery Area**

|  |  |  |  |  |  |  |
| --- | --- | --- | --- | --- | --- | --- |
|  | **(1)** | | **(2)** | | **(3)** | |
| Interior Health |  | [95% C.I.] |  | [95% C.I.] |  | [95% C.I.] |
| *East Kootenay* | 0.513 | [0.021, 1.005] | 4.362 | [3.611, 5.113] | 197 | [127.8, 266.2] |
| *Kootenay Boundary* | 0.216 | [0.094, 0.338] | 4.447 | [3.79, 5.104] | 155 | [132.9, 177] |
| *Okanagan* | 0.311 | [0.238, 0.384] | 4.954 | [4.631, 5.277] | 138.4 | [129, 147.8] |
| *Thompson Cariboo* | 0.317 | [0.211, 0.423] | 4.745 | [4.294, 5.196] | 150.4 | [134, 166.8] |
| Fraser Health |  |  |  |  |  |  |
| *Fraser East* | 0.436 | [0.311, 0.561] | 4.892 | [4.504, 5.28] | 154.2 | [140.8, 167.6] |
| *Fraser North* | 0.272 | [0.215, 0.329] | 4.135 | [3.953, 4.317] | 180.9 | [173.2, 188.7] |
| *Fraser South* | 0.479 | [0.397, 0.561] | 5.294 | [4.998, 5.59] | 151.5 | [142.1, 160.8] |
| Vancouver Coastal Health |  |  |  |  |  |  |
| *Richmond* | 0.402 | [0.204, 0.6] | 5.093 | [4.454, 5.732] | 146.1 | [126, 166.1] |
| *Vancouver* | 0.457 | [0.426, 0.488] | 5.079 | [4.993, 5.165] | 161 | [157.1, 164.8] |
| *North Shore/Coast Garibaldi* | 0.348 | [0.246, 0.45] | 5.728 | [5.24, 6.216] | 156.4 | [143, 169.8] |
| Island Health |  |  |  |  |  |  |
| *South Vancouver Island* | 0.379 | [0.305, 0.453] | 5.079 | [4.82, 5.338] | 160.2 | [151, 169.3] |
| *Central Vancouver Island* | 0.409 | [0.291, 0.527] | 5.866 | [5.431, 6.301] | 143.2 | [131.4, 154.9] |
| *North Vancouver Island* | 0.28 | [0.188, 0.372] | 5.472 | [4.686, 6.258] | 139.4 | [120.2, 158.7] |
| Northern Health |  |  |  |  |  |  |
| *Northwest* | 0.401 | [0.227, 0.575] | 5.174 | [4.523, 5.825] | 141.2 | [97.8, 184.6] |
| *Northern Interior* | 0.462 | [0.344, 0.58] | 3.647 | [3.341, 3.953] | 132.4 | [111.9, 152.9] |
| *Northeast* | 0.388 | [0.178, 0.598] | 4.322 | [3.581, 5.063] | 102.7 | [72.7, 132.8] |
| *(1) - Adjusted hospitalization days per person-quarter; **(2) - Adjusted physician billing days per person-quarter; ***(3) - Adjusted non-ART prescription drug days per person-quarter Covariate adjustment for all models included: age, gender, era of diagnosis, calendar year, moved in past 12 months, ART-status, CD4 cell count, pVL, chronic disease score, Charlson comorbidity index, HIV risk group. | | | | | | |
|  |  |  |  |  |  |  |
|  |  |  |  |  |  |  |
|  |  |  |  |  |  |  |

**Table S4. Sensitivity analysis for adjusted total quarterly medical care costs per person-quarter by Health Service Delivery Area**

|  |  |  |  |  |  |  |
| --- | --- | --- | --- | --- | --- | --- |
|  | **(1)** | | **(2)** | | **(3)** | |
| Interior Health |  | [95% C.I.] |  | [95% C.I.] |  | [95% C.I.] |
| *East Kootenay* | $4912 | [$2636, $7188] | $4924 | [$2631, $7217] | $4937 | [$2462, $7412] |
| *Kootenay Boundary* | $4048 | [$3580, $4516] | $4047 | [$3581, $4513] | $3916 | [$3477, $4355] |
| *Okanagan* | $4274 | [$3976, $4572] | $4239 | [$3947, $4531] | $4261 | [$3969, $4553] |
| *Thompson Cariboo* | $3775 | [$3407, $4143] | $3779 | [$3407, $4151] | $3722 | [$3348, $4096] |
| Fraser Health |  |  |  |  |  |  |
| *Fraser East* | $4204 | [$3749, $4659] | $4208 | [$3751, $4665] | $4170 | [$3707, $4633] |
| *Fraser North* | $4170 | [$3980, $4360] | $4164 | [$3974, $4354] | $4177 | [$3932, $4422] |
| *Fraser South* | $4677 | [$4375, $4979] | $4679 | [$4375, $4983] | $4529 | [$4231, $4827] |
| Vancouver Coastal Health |  |  |  |  |  |  |
| *Richmond* | $4028 | [$3624, $4432] | $4023 | [$3619, $4427] | $4066 | [$3649, $4483] |
| *Vancouver* | $4722 | [$4630, $4814] | $4702 | [$4612, $4792] | $4712 | [$4624, $4800] |
| *North Shore/Coast Garibaldi* | $4245 | [$3871, $4619] | $4234 | [$3862, $4606] | $4290 | [$3908, $4672] |
| Island Health |  |  |  |  |  |  |
| *South Vancouver Island* | $4294 | [$4110, $4478] | $4289 | [$4105, $4473] | $4303 | [$4117, $4489] |
| *Central Vancouver Island* | $4346 | [$4068, $4624] | $4344 | [$4068, $4620] | $4398 | [$4092, $4704] |
| *North Vancouver Island* | $3975 | [$3538, $4412] | $3982 | [$3543, $4421] | $3925 | [$3476, $4374] |
| Northern Health |  |  |  |  |  |  |
| *Northwest* | $3989 | [$3377, $4601] | $3978 | [$3366, $4590] | $3798 | [$3181, $4415] |
| *Northern Interior* | $4184 | [$3751, $4617] | $4192 | [$3755, $4629] | $4295 | [$3876, $4714] |
| *Northeast* | $4311 | [$3151, $5471] | $3909 | [$3182, $4636] | $4221 | [$2990, $5452] |
| *(1) - Baseline model including all costs; **(2) - Sensitivity analysis excluding PHSA costs; ***(3) - Sensitivity analysis using highest-cost HSDA per person-quarter Covariate adjustment for all models included: age, gender, era of diagnosis, calendar year, moved in past 12 months, ART-status, CD4 cell count, pVL, chronic disease score, Charlson comorbidity index, HIV risk group. | | | | | | |
|  |  |  |  |  |  |  |
|  |  |  |  |  |  |  |
|  |  |  |  |  |  |  |

**Figure S1. British Columbia Health Service Delivery Areas**

**
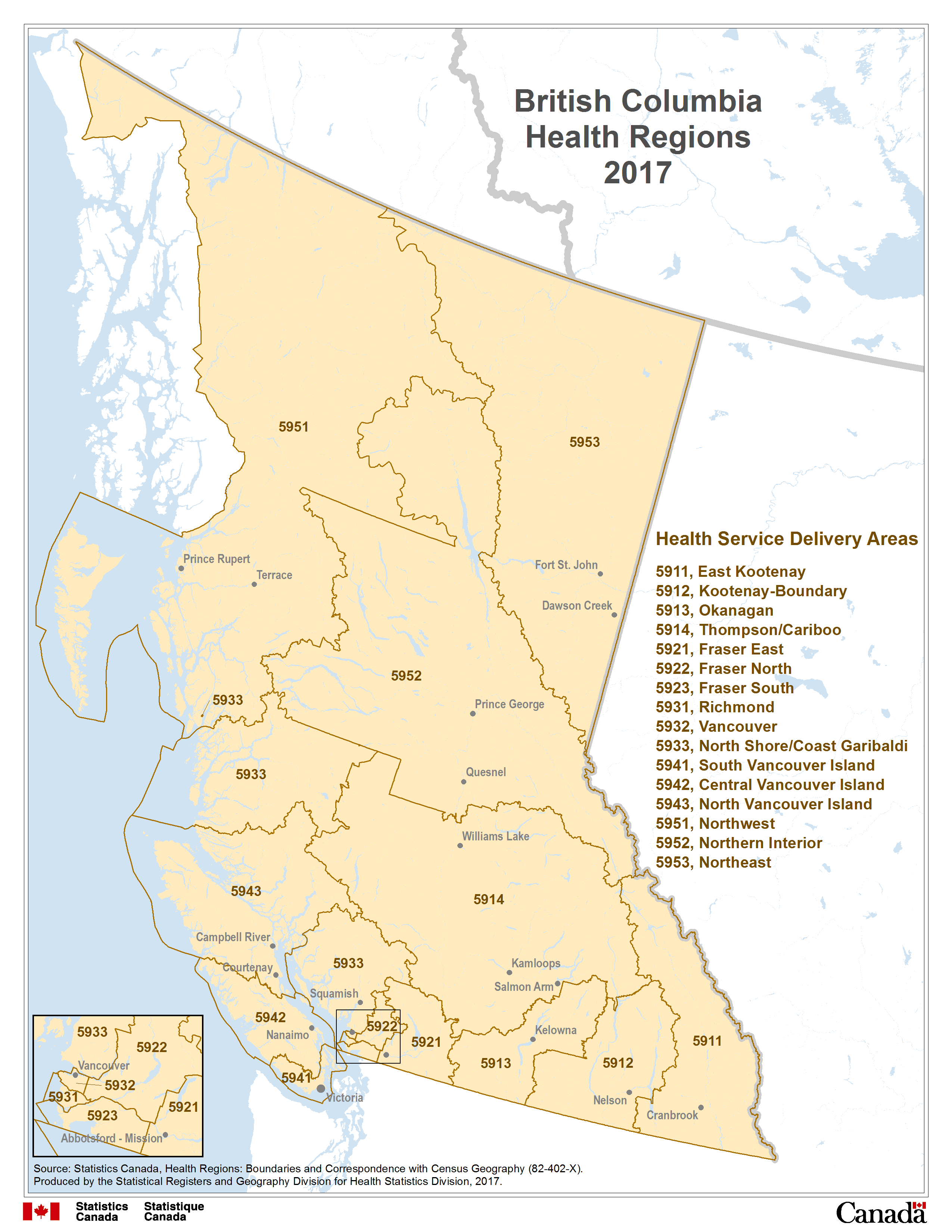
**

Source: Statistics Canada, British Columbia Health Regions 2017, July 4, 2019 [[1](#_ENREF_1)]. Reproduced and distributed on an "as is" basis with the permission of Statistics Canada.

**References**

1. **British Columbia Health Regions, 2017** [https://www150.statcan.gc.ca/n1/pub/82-402-x/2017001/maps-cartes/rm-cr12-eng.htm [Accessed July 4, 2019]]
